# The Relationship between Sense of Belonging and Well-Being Outcomes in Emerging Adults with Care Experience

**DOI:** 10.3390/ijerph20136311

**Published:** 2023-07-07

**Authors:** Amanda Hiles Howard, Getrude Dadirai Gwenzi, Lindsey Newsom, Belay T. Gebru, Nicole Gilbertson Wilke

**Affiliations:** 1 Department of Psychology, Samford University, 800 Lakeshore Dr., Homewood, AL 35229, USA; 2 Department of Social Work, University of Zimbabwe, 630 Churchill Avenue, Harare, Zimbabwe; 3 Hope for the Fatherless, 558 Castle Pines Parkway, Castle Pines, CO 80108, USA; 4 CAFO Center on Applied Research for Vulnerable Children and Families, 505 Los Arces Monteflor II Cayma, Arequipa, Peru

**Keywords:** belonging, care leavers, alternative care, social resources, emerging adulthood

## Abstract

Robust social resources that lead to a healthy sense of belonging are imperative during emerging adulthood. However, young adults with alternative care experience, such as residential or foster care, often lack the social resources necessary to transition to adulthood successfully. Though some research has examined the importance of social resources in this population, less is known about a sense of belonging, which is associated with these social resources. The current study examined the association between care experience, belonging, and well-being outcomes among emerging adults (n = 703) who were separated from their biological parents during care and received alternative care in 16 nations. The presence of belonging was associated with type of alternative care, age at separation, and reason for separation. There was also an association between belonging and well-being outcomes, including homelessness and suicidal ideation. Adults lacking a sense of belonging reported higher rates of homelessness and suicidal ideation, lower life satisfaction, and lower resilience. The study has global implications for enhancing social support for emerging adults with care experience in order to facilitate a sense of belonging as a social resource.

## 1. Introduction

During emerging adulthood [1], individuals undergo developmental changes the require significant social support. Emerging adulthood has been defined as the second and third decades of life. Many studies of this population sample individuals beginning at age 18 and moving into the late twenties [2,3,4]. Further, emerging adulthood bears potential for positive change for young people [5]. Successful outcomes during this stage include gainful employment, stable housing and relationships, and other markers of resilience [6,7]. Having a sense of belonging, adequate social resources, and connection to a community has been linked with positive transitions into adulthood [8]. Belonging is defined as a fundamental human need encompassing lasting, stable, and predominantly positive interpersonal relationships [9]. A sense of belonging is an important protective factor for emerging adults and contributes to coping with stress, positive adjustment while transitioning to adulthood, and better overall outcomes [10,11]. The absence of belonging is, thus, associated with negative outcomes, including poor mental health and lower resilience [11,12].

A multitude of factors, such as poverty, parental death, and maltreatment, contribute to the separation of millions of children and youth from biological parental care during their childhood worldwide [13,14]. Throughout these separations, young people frequently reside in alternative care settings, including residential facilities, kinship care arrangements, and foster care placements [15]. In addition to biological parents, these youth are often separated from their siblings, friends, and communities of origin [16]. Young adults who experienced separation from parental care during childhood are more likely to experience negative outcomes in adulthood including a higher risk of self-harm [17], violent criminal behavior [18], and homelessness [19]. Previous studies have also shown evidence of poor mental health, lower life satisfaction, insecure attachment, and less resilience among young adults with care experience due to their limited social connections [12,20,21]. Specifically, adults with care experience have been documented to experience challenges during emerging adulthood due to strained relationships with their biological families [22], poor social networks [16], and lack of social capital and resources [10,23]. Evidence suggests that being separated from parental care deprives individuals of the formal and informal social resources that are important for a sense of belonging and support that comes from interpersonal relationships with family, friends, and community [16,24].

Some research has examined the importance of a robust sense of belonging among individuals with care experience [10]. Studies have found evidence of some youth with care experience having little or no sense of belonging to a family [25]. Their narratives of belonging are often fragile and continue to be weakened by their weak social connections [24]. Correspondingly, adults with care experience often find themselves seeking to create a sense of belonging through friends, starting their own families, and partners [22]. This work demonstrates the importance that a sense of belonging may play in the lives of individuals with care experience. However, our understanding of the association between belonging and care experience remains limited. Specifically, little is known about the relationship between sense of belonging during separation from parental care and the ways it impacts later well-being outcomes.

### The Present Study

The present study has three objectives. First, examining how care experiences are associated with a sense of belonging during separation from parental care. Second, investigating the connection between a sense of belonging and homelessness and suicidal ideation among emerging adults. Finally, understanding how a sense of belonging while separated from parental care is related to life satisfaction and resilience in emerging adulthood.

## 2. Material and Methods

The data presented were gathered as part of a broader initiative that explored healthy development of adults with care experience. The current sample is a subset of measures and participants from the larger research project. Specifically, it includes participants who resided in nations during childhood where there were more than 15 participants, and who were under the age of 30 years old.

### 2.1. Participants

Participants were 703 emerging adults who were separated from parental care during childhood. Participants resided in 16 nations at the time of the survey including India (n = 129), Poland (n = 94), Kenya (n = 77), and the United States of America (n = 67). Participants ranged in age from 18–29 (M = 22.61; SD = 3.30) and approximately half (54.9%) were male. More detailed demographic information can be found in Table 1.

### 2.2. Measures

General Demographics. The participants provided information on various aspects of demographic details, including current age, country of residence, gender, education level, marital status, parental status, employment status, experiences of homelessness in adulthood, and suicidal ideation.

Care Experience Demographics. The participants responded to multiple items that gathered demographic information specific to their experiences of being separated from their biological parents during childhood. These items included the country where the separation occurred, the age at which the separation took place, the reasons behind the separation, the types of placements involved, and the number of placements experienced. It is important to note that many adults with care experience report multiple reasons for their initial separation from their biological parents, as well as experiencing various types of placements during their separation. To comprehensively capture these experiences, the questions were presented in a “check-all-that-apply” format. Additionally, the Human Development Index (HDI) [26] for the country of residence during childhood was included as a demographic variable. The HDI is a composite indicator that assesses the well-being of individuals in a given country, taking into account factors such as life expectancy, education level, and per capita income. The inclusion of HDI in the current study is significant as it captures national-level differences among participants.

Care Experience Survey. Participants were asked about their experiences during their time in care and how they perceived those experiences to have influenced them as adults. Regarding sense of belonging, participants were presented with the question, “As you reflect on your time in care as a child, did you ever experience a place or time where you felt a sense of belonging?” This question consisted of two parts: (1) a multiple-choice response (no or yes) and (2) an open-ended section allowing participants to provide further information. However, only the data from the multiple-choice response (no/yes) is included in the current study. Among the participants, 57.3% (n = 403) reported feeling a sense of belonging at some point during their time in care, while 42.7% (n = 300) indicated that they did not feel a sense of belonging.

Brief Resilience Scale. The measurement of resilience utilized the Brief Resilience Scale [27], consisting of six items that assess resilience on a Likert-like scale ranging from 1 (strongly disagree) to 5 (strongly agree). Higher scores on the scale indicate greater levels of resilience. The internal consistency of the scale, as indicated by Cronbach’s Alpha, was 0.717.

Satisfaction with Life Scale. The Satisfaction with Life Scale is a questionnaire consisting of five items intended to assess an individual’s level of life satisfaction [28]. All items are rated on a 7-point Likert-like scale, ranging from 1 (strongly disagree) to 7 (strongly agree). Higher scores on the scale indicate a greater level of life satisfaction. The possible range of scores is between 5 and 35, with a score of 20 representing a neutral or average point on the scale. Scores falling between 5 and 9 indicate a high level of dissatisfaction with life, while scores between 31 and 35 suggest an individual is highly satisfied with life. The internal consistency of the scale, as measured by Cronbach’s Alpha, was 0.805.

### 2.3. Translations

The survey items were translated from English into Hindi, Spanish, and Thai languages. The translation process adhered closely to the cross-cultural research methodology guidelines outlined by Brislin [29]. To ensure accuracy, independent blind back-translations, and educated translations were conducted for all survey questions. Two bilingual content experts performed the translation for each language, and at least one of the translators was a native speaker of that language. Discrepancies between translators were minimal, and any differences were resolved through consensus reached via discussions among the translators.

### 2.4. Procedure

This study received ethical approval from the Institutional Review Board of the author’s university. The recruitment process employed a combination of convenience and chain-referral sampling methods. Recruitment notifications were posted on relevant organizations’ websites and sent via email to their mailing lists. Furthermore, the announcements were circulated through chain referral sampling. All data collection measures were administered online using a survey platform, with the questionnaires presented in a randomized order. Informed consent was obtained from participants at the beginning of the survey by checking a box to indicate their agreement. The completion rate for the study was 74.0%.

### 2.5. Statistical Analysis

The primary independent variable was sense of belonging, which was a categorical variable belonging (0 = no sense of belonging while separated from parental care; 1 = sense of belonging while separated from parental care). Categorical dependent variables included adult homelessness and suicidal ideation. Continuous dependent variables included resilience and life satisfaction. Pearson’s chi-square (χ2) tests were conducted to examine the relationships between categorical variables, such as gender and sense of belonging. Cramer’s V was used to measure effect size for the chi-square test of independence. Independent sample *t*-tests were conducted to assess any differences between categorical independent variables (i.e., belonging) and continuous variables, such as current age. Multivariate analysis of variance tests (MANOVAs) were performed to examine the relationships between multiple continuous variables (ex. age at separation, HDI, etc.) and the categorical independent variable (i.e., sense of belonging). This was used to decrease the likelihood of Type I Error. Wilks’ lambda was used to measure how well the independent variable discriminated between different groups in the MANOVA. Analysis of Covariance (ANCOVA) was used to test the main effects of the categorical independent variable (i.e., belonging) on continuous dependent variables (i.e., resilience and life satisfaction), while controlling for the effects of continuous demographic variables (i.e., HDI of nation of residence during childhood).

## 3. Results

The Results section is organized in four sections corresponding to the aims of the present study. The first section examines descriptive statistics for the sample, including care experience variables. The next section examines the relationships between sense of belonging and care experiences, as well as other demographic variables. The third section explores the relationship between sense of belonging and adult homelessness and suicidal ideation. The final section investigates the associations between sense of belonging and resilience and life satisfaction while accounting for demographic variables.

### 3.1. Descriptive Statistics

Participants lived in 16 countries during childhood. The most frequently reported countries of childhood residence were India (n = 130), Kenya (n = 103), Poland (n = 97), and Zimbabwe (n = 55). Table 2 presents the frequencies and percentages of the nations where participants resided during their childhood. The participant’s age at separation from parental care ranged from 0–16 (M = 7.98; SD = 5.06). Participants indicated reasons that led to their separation from parental care (See Table 3), with the most frequent reasons being death of a parent(s) (29.9%), abandonment/relinquishment (23.2%), family stress/instability (20.6%), and poverty (19.2%). Approximately 54.1% of participants indicated experiencing more than one placement following their separation from parental care. The total number of placements reported by participants varied from 1 to 16, with an average of 2.09 (SD = 1.80). Participants also disclosed different types of placements, as shown in Table 3. The most commonly reported types of placements were residential/group care (49.5%), kinship placement (37.7%), and foster care (20.9%).

### 3.2. Belonging and Descriptive Statistics

To investigate the associations between demographic variables and the sense of belonging in emerging adults with care experience, a series of analyses were performed. Pearson’s chi-square (χ2) tests were utilized to examine the relationships between categorical demographic variables (such as gender, marital status, employment status, and having children) and the classification of belonging (0 = no sense of belonging while separated from parental care; 1 = sense of belonging while separated from parental care). Furthermore, an independent sample *t*-test was conducted to assess any differences between the sense of belonging categories and the participants’ current age. The results indicated no significant differences in these analyses.

To explore the associations between care experience variables and sense of belonging among emerging adults, a series of analyses were performed. Pearson’s chi-square (χ2) tests were used to analyze the relationships between categorical care experience variables, including the reason for separation and types of care, and sense of belonging. For the reason for separation variables, results revealed significant differences in sense of belonging classification for abandonment/voluntary relinquishment (χ2 (2) = 4.35, Cramer’s V = 0.15), education (χ2 (2) = 4.28, Cramer’s V = 0.08), and maltreatment (χ2 (2) = 15.43, Cramer’s V = 0.15). Of participants separated due to abandonment/voluntary relinquishment, 42.8% reported a sense of belonging. Of participants separated due to maltreatment, 36.2% reported a sense of belonging. By contrast, 65.7% of participants separated due to education reported a sense of belonging during their separation. Regarding types of placements during separation, results revealed significant differences in sense of belonging for residential care (χ2 (2) = 7.53, Cramer’s V = 0.10), reintegration (χ2 (2) = 5.32, Cramer’s V = 0.09), and kinship care (χ2 (2) = 8.10, Cramer’s V = 0.11). Of participants who experienced residential care, 62.5% reported a sense of belonging. Of participants who experienced reintegration to biological parents, 67.5% reported a sense of belonging. Of participants who experienced kinship care, 64.2% reported a sense of belonging. Participants who had experienced residential care, reintegration, or kinship care were found to be more likely to report a sense of belonging during their separation compared to those who had been in other types of care settings. The remaining variables were non-significant (see Table 3).

A MANOVA was performed to investigate the relationships between continuous care experience variables and Human Development Index (HDI) of the nation of residence during childhood, and the sense of belonging. The results of the MANOVA indicated a significant effect, Wilks’ Lambda = 0.998, *p* < 0.001. Data revealed that sense of belonging category was significantly related to age at separation from parental care, F (1, 702) = 4.37, *p* < 0.05, ŋ^2^ = 0.006. Findings suggested that participants that did not have sense of belonging were significantly younger when separated from their biological parents (M = 7.52; SD = 5.05) than those who felt a sense of belonging (M = 8.32; SD = 5.04). No other significant differences were found.

### 3.3. Belonging and Adult Outcomes

Belonging, Homelessness, and Suicidal Ideation. To explore the relationships between homelessness, suicidal ideation, and the sense of belonging classification among emerging adults, a series of analyses were conducted. The current sample reported high rates of adult homelessness (14.2%; n = 100) and suicidal ideation (54.8%; n = 385). Pearson’s chi-square (χ2) tests were utilized to examine the relationships between homelessness, suicidal ideation, and sense of belonging classification. Frequencies for sense of belonging by adult homelessness and suicidal ideation can be found in Table 4. Results revealed significant differences in sense of belonging for adult homelessness (χ2 (2) = 28.20, Cramer’s V = 0.20) and suicidal ideation (χ2 (2) = 25.88, Cramer’s V = 0.19). Among the emerging adults who reported experiencing homelessness, 67.0% indicated that they had never felt a sense of belonging during their separation from parental care. Similarly, among those who had considered suicide, 67.9% reported that they had never experienced a sense of belonging while in care.

Belonging and Resilience. To explore the relationships between sense of belonging during separation from parental care and resilience in emerging adulthood, an ANCOVA was performed. The covariates included in the analysis were current age, age at separation, and the HDI score for the nation of residence during childhood. As can be seen in Table 5, data revealed a significant main effect for belonging F(1, 702) = 16.71, *p* < 0.001, ŋ^2^ = 0.023. Emerging adults who did not have a sense of belonging during their separation from parental care had significantly lower resilience (M = 3.09; SD = 0.73) than those who felt they belonged (M = 3.31; SD = 0.71).

Belonging and Life Satisfaction. To investigate the relationships between sense of belonging while separated from parental care and life satisfaction during emerging adulthood, an ANCOVA was conducted. The covariates included in the analysis were current age, age at separation, and the HDI score for the nation of residence during childhood. Data revealed a significant main effect for belonging, F(1, 702) = 43.97, *p* < 0.001, ŋ^2^ = 0.059 (See Table 5). Emerging adults who did not have a sense of belonging during their separation from parental care had significantly lower satisfaction with life (M = 18.81; SD = 6.76) than participants who felt they belonged (M = 22.20; SD = 6.65).

## 4. Discussion

The study examined the relationship between (1) sense of belonging when separated from parental care during childhood and care experiences, (2) adult homelessness and suicidal ideation, and (3) life satisfaction and resilience in a sample of emerging adults with care experience from multiple nations. The findings in the present study show that a sense of belonging is influenced by different types of care experiences, including reasons for placement in care, age at separation, and type of alternative care. The study also found negative associations between sense of belonging and homelessness and suicidal ideation, along with positive associations between sense of belonging and life satisfaction and resilience.

Separation from parental care puts children at risk of losing their sense of belonging, crucial for overall development. This study revealed that reasons for separation from parental care significantly impacted the sense of belonging among these individuals. In particular, adults who were abandoned and maltreated as children had a lower sense of belonging. Early maltreatment has long been associated with negative effects in children, including issues with attachment, communication, and social deficits [30]. Likewise, the type of alternative care placement following separation from parental care impacted the sense of belonging among adults with care experience. Individuals placed in residential care, kinship care, or reunited with their biological parents reported a sense of belonging, unlike those in other care settings like foster care and adoption. Previous research has emphasized that belonging, especially within family relationships, is not a static achievement but an ongoing process that can be fostered through dynamic practices [8]. For adults with care experience who resided in residential care, kinship care, and who were integrated with their biological families, a sense of belonging may have developed through the family practices they experienced whilst in care. These family practices have been found to assist in developing a sense of belonging [31].

Previous studies have shown homelessness among care leavers can be attributed to a lack of social support or family members who can accommodate them [32]. In the present study, not having a sense of belonging was linked with homelessness. In line with previous studies, lack of belonging among young adults with care experience may have stemmed from the absence of a sense of family [22,33]. “Family” for individuals with care experience has been described as “a place of belonging, unconditional love, acceptance, understanding and support” [34] (p. 250). In its absence, several negative outcomes have been documented, including homelessness, as found in the present study [11,31]. Moreover, the current study revealed a significant association between a lower sense of belonging and higher levels of suicidal ideation. Suicidal ideation is known to be associated with adverse mental health outcomes, including anxiety and depression, which are negatively correlated with life satisfaction [6]. Kelly et al. [35] found high rates of suicide attempts in the preceding year among a sample of care leavers in Northern Ireland, which is evidence of low mental health. The present study found a positive association between having a sense of belonging and higher levels of life satisfaction, in contrast to not having a sense of belonging. This implies that having social support, which creates a sense of belonging, will in turn have positive mental health outcomes for emerging adults with care experience. In their theoretical work regarding family, McCarthy [31] noted that a higher sense of belonging has been associated with higher levels of life satisfaction and well-being. Lastly, the present study revealed a positive relationship between the presence of a sense of belonging and resilience. Adults with a sense of belonging were found to have higher resilience levels. This supports findings by Sulimani-Aidan and Tayri-Schwartz [10] who also found a robust and direct association among Israeli care leaver’s sense of belonging and resilience.

The current results are important for several reasons. Firstly, the study underscores the significance of having a sense of belonging for adults who grew up separated from parental care. The lack of belonging affects life satisfaction and resilience, especially during their transition into adulthood. The study adds to the existing body of literature on the subjective well-being of emerging adults with care experience, addressing a gap in the current research. Knowledge about the factors that impact life satisfaction of emerging adults with care experience could contribute to positive outcomes. Belonging is also a significant factor for the positive development of adults with care experience, and knowledge about the factors that reduce their sense of belonging will contribute to efforts to improve their social resources and important sources of belonging such as their interpersonal relationships.

### 4.1. Recommendations for Practice

Practitioners engaged in caring for children separated from parental care should be aware of the importance of a sense of belonging in this population, and associations with mental health and well-being outcomes. Although mechanisms for facilitating belonging were outside the scope of this investigation, it is likely that practitioners support a sense of belonging in children in care simply by asking the children what actions or experiences contribute to their sense of belonging. This practice should not only be considered for children in care, but also for adults who were separated from parental care as children, as they may be more likely to struggle with the concept of belonging.

### 4.2. Limitations and Future Research

The study acknowledges several limitations that should be taken into account when interpreting the findings. The methodology employed convenience and chain referral sampling methods, which may limit the generalizability of the results. Additionally, the research relied on a one-time, online survey, and the survey was only translated into a limited number of languages. Participants in the study were required to have internet access and literacy skills, potentially biasing the sample towards individuals with higher education levels and socioeconomic status, as well as more positive long-term outcomes. These factors raise concerns about the representativeness of the sample. Future research should address these limitations by employing more robust sampling methods, expanding the language options, and aiming for more diverse and representative samples that encompass the experiences of all adults with alternative care backgrounds.

We also did not collect objective measures of socioeconomic status or and subjective measures of wealth for individual participants. These variables may influence protective factors and life satisfaction. It is crucial to acknowledge that different types of alternative care settings can often arise within distinct household environments. These environments may be influenced by objective measures of socioeconomic status, leading to variations in the quality and type of alternative care available. Certain types of alternative care settings may be more prevalent in economically disadvantaged communities, while others may be more commonly found in more affluent settings. This socioeconomic disparity can impact the resources, support systems, and overall living conditions provided within these alternative care arrangements, which in turn influence protective factors. Therefore, it is important to consider objective measures of socioeconomic status when examining the characteristics and outcomes associated with different types of alternative care, as they play a significant role in shaping the environment in which care is provided. Further, subjective measures of wealth may also influence well-being outcomes. Indeed, one’s perception of wealth or poverty can be subjective and influenced by various factors, including their upbringing, social environment, and personal experiences. An individual who was raised in an affluent community may compare themselves to their wealthy peers and perceive themselves as comparatively less wealthy, despite objectively having a high level of wealth. Conversely, someone from a less affluent background may compare themselves to those in even more disadvantaged circumstances and perceive themselves as relatively better off, despite objectively having less wealth. It is important to recognize that wealth and poverty encompass not only material possessions but also subjective interpretations and individual perspectives. These subjective assessments of wealth can influence perception of overall well-being and life satisfaction. Future research should include both objective and subjective measures of socioeconomic status and wealth to address these concerns and potential sampling bias.

## 5. Conclusions

The current study highlights the long-term importance of facilitating a sense of belonging among children separated from parental care. Participants who had a sense of belonging while in care had better outcomes than those that did not. The study’s findings highlight the significant relationship between resilience and a sense of belonging among emerging adults with care experience, indicating the need for enhanced efforts to strengthen social resources and networks for this population. This implies that interventions and policies should focus on fostering a sense of belonging and providing adequate social resources to promote the resilience of individuals with care experience. Study findings also suggest that the separation experience has an impact on some adulthood indicators which will have an influence on child welfare policy and practice. To address the negative long-term impacts experienced by individuals, measures should be implemented to minimize the separation of children and young people from their parents. Research has demonstrated that such separations can have detrimental effects that extend into adulthood. Therefore, it is crucial to implement strategies aimed at preventing unnecessary separations and promoting family preservation whenever possible. These measures include family preservation services, reintegration services which contribute to creating social and community networks which provide care leavers with a sense of belonging, and improving their mental and physical outcomes, such as access to employment and accommodation.

## Figures and Tables

**Table 1:** Participant Demographic (*N* = 703).

		*F*	%
Gender			
	Female	317	45.1
	Male	386	54.9
Education			
	Less than high school degree	79	11.2
	High school diploma or equivalent	271	38.5
	Some college but no degree	146	20.8
	Associate degree/Trade	47	6.7
	Bachelor’s degree	135	19.2
	Master’s degree	19	2.7
	Doctoral degree/professional	6	0.9
Employment			
	Not working (looking for work)	169	24.0
	Not working (other)	60	8.5
	Working (paid employee)	289	41.1
	Full-time student	185	26.4
Marital Status			
	Never married	549	78.1
	Married	71	10.1
	Divorced/separated	16	2.3
	Other	67	9.5
Children			
	No	627	89.2
	Yes	76	10.8

**Table 2:** Frequency, Percentage, and Human Development Index for Nation of Residence During Childhood for Nations (*N* = 703).

Nation	*F*	%	HDI (Human Development Index)
India	130	18.5	0.645
Kenya	103	14.7	0.601
Poland	97	13.8	0.601
Zimbabwe	55	7.8	0.571
United States of America	52	7.4	0.926
Portugal	41	5.8	0.864
Mexico	35	5.0	0.779
Uganda	34	4.8	0.544
Rwanda	26	3.7	0.543
Demo. Republic of the Congo	25	3.6	0.480
Ethiopia	22	3.1	0.485
Thailand	19	2.7	0.777
Peru	17	2.4	0.777
China	16	2.3	0.761
Romania	16	2.3	0.828
South Africa	15	1.7	0.709

**Table 3:** Frequencies and Percentages for Reason for Separation and Types of Placements (*N* = 703).

Category		*F*	%	Belonging %
Reason for Separation				
	Parental Death	210	29.9	59.5
	Abandonment/Voluntary Relinquishment	145	20.6	42.8 *
	Family Stress/Instability	143	20.3	51.7
	Poverty	135	19.2	53.3
	Education	120	17.1	65.7 *
	Maltreatment	80	11.4	36.2 *
	Lack of Parenting Skills	94	13.4	57.4
	Parental Marriage/Divorce	65	9.2	60.1
	Substance Abuse	52	7.4	48.1
	Unplanned Pregnancy	19	2.7	55.4
	Forced Displacement	42	6.0	61.9
	Parental Imprisonment	38	5.4	57.9
	Caregiver Health	40	5.7	47.5
	Participant chose to leave	32	4.6	59.4
Types of Placements During Separation				
	Residential Care	348	49.5	62.5 *
	Kinship Care	265	37.7	64.2 *
	Foster Care	147	20.9	51.9
	Reintegration	114	16.2	67.5 *
	Domestic Adoption	56	8.0	55.3
	Independent Living (>18)	102	14.5	56.9
	Homelessness Without Caregiver	46	6.5	58.3
	International Adoption	34	4.8	50.5

* *p* < 0.05.

**Table 4:** Frequencies for Sense of Belonging by Adult Homelessness and Suicidal Ideation (*N* = 703).

	Did Not Belong		Belonged		χ^2^	Cramer’s *V*
	*F*	%	*F*	%		
Homelessness					28.20 *	0.20
No	233	38.6	370	61.4		
Yes	67	67.0	33	33.0		
Considered Suicide					25.88 *	0.19
No	39	12.3	279	87.7		
Yes	261	67.9	124	32.1		

* *p* < 0.001.

**Table 5:** Means and Standard Deviations for Life Satisfaction and Resilience by Sense of Belonging (*N* = 703).

Category	M	SD	*F*	η^2^_p_
Resilience			16.71 *	0.023
No Belonging	3.09	0.73		
Belonging	3.31	0.71		
Life Satisfaction			43.97 *	0.059
No Belonging	18.81	6.76		
Belonging	22.20	6.65		

* *p* < 0.001.

## Data Availability

The corresponding author can be contacted for requesting access to the data presented in this study. The data are not publicly available in order to maintain participant confidentiality.
